# Behavior of a Two-Way Lightweight Steel–Concrete Composite Slab Voided with Thin-Walled Core Boxes towards Sustainable Construction

**DOI:** 10.3390/ma13184129

**Published:** 2020-09-17

**Authors:** Yongqi Huang, Vincent J.L. Gan, Huaguo Chen, Jianjun Yang

**Affiliations:** 1 School of Civil Engineering, Central South University, Changsha 410075, China; yancy7@csu.edu.cn; 2 National Engineering Laboratory for High-Speed Railway Construction, Changsha 410075, China; 3 Department of Building, School of Design and Environment, National University of Singapore, Singapore 117566, Singapore; vincent.gan@nus.edu.sg; 4 Department of Architecture and Civil Engineering, City University of Hong Kong, Hong Kong 999077, China

**Keywords:** flexural performance, steel–concrete composite slab, thin-walled core boxes, sustainable construction, membrane effect

## Abstract

High-performance engineered structural systems are crucial for sustainable development in the field of construction. In our previous research, a novel steel–concrete composite beam with transverse and longitudinal hidden girders exhibited good flexural behavior and desirable ductility. However, there is a dearth of studies on the flexural response of a steel–concrete composite slab voided with thin-walled core boxes. Therefore, in this study, we investigated the overarching flexural mechanism of the proposed structure when subjected to uniform vertical loads. The experimental detection results illustrated that the deflection value of the composite beam was 95.75% less than the GB/T 50152-2012 recommendation. Numerical results further validated this observation. The recorded data from the strain profile at the mid-span of the frame girder indicated that there was a considerable membrane effect, which delayed the strain growth of rebars, yielding appreciable bearing capacity. Thus, two original approaches to predicting the ultimate load of this novel structure are proposed, considering limit analysis using the upper-bound method and the membrane effect, with the latter closely linked to the experimental results. The findings can promote the extensive application of similar sustainable systems and inspire further advancements in advanced engineering structures.

## 1. Introduction

During the 12th Five-Year Plan period (2011–2015), China set mandatory energy conservation and CO_2_ intensity reduction targets, propelling the government to accelerate industrialized construction in an environmentally friendly manner [1,2]. The recent boom in new construction has substantially increased the demand for energy and various resources [3]. The reinforcement concrete (RC) structure is one of the most commonly used building forms, contributing to the consumption of considerable amounts of material such as cement, concrete, and reinforcing steel, which in turn extensively impacts the built environment and China’s sustainable development agenda [4,5,6]. Thus, it is imperative to explore new high-performance structural systems that enhance the utilization rate of structural building materials and improve compliance with the requirements for green buildings [7,8,9].

In this regard, utilizing highly efficient hollow–core slabs (HCS) for the floor or roof is a workable approach to residential, commercial, and industrial projects in low-seismic areas [10]. Furthermore, traditional non-prestressed HCS contains void fillers in diverse shapes (e.g., spherical, cylindrical, or cubical) along its span to reduce self-weight by eliminating the cross-sectional concrete volume, which is structurally insignificant relative to flexural resistance, thereby enabling longer spans with a relatively shallow thickness [11,12,13,14]. When such a structure is projected as a horizontal component, its flexural performance is critical under uniformly distributed loads. In recent years, many studies on mechanical behavior have been conducted on cast-in-situ two-way voided slabs and other composite structures, with the essential goal of applying environmental systems to residential buildings [15,16,17,18,19,20,21,22,23]. These analytical, numerical, and experimental studies compared with studies on traditional hollow slabs (reinforced concrete waffle slabs and cellular flat plates), show significant demonstrable improvements in structural performance, void filler material, and cost savings. Consequently, the voided slab system has been adopted in building design units and by technicians for its advantages of low concrete consumption, considerable floor-to-floor height, and expeditious construction with a high degree of quality control [24]. However, for engineers to employ this system in the blueprint of an entire building in practical engineering solutions, an in-depth understanding of the structural performance of the slab across the broad structure is required, including the floor, beams, and columns. Furthermore, although steel–concrete composite beams lower human resource and construction materials cost and reduce CO_2_ emissions, there is presently limited knowledge on combining HCS with steel girders, impeding the development of this novel environmental-structural system. To achieve composite structural integrity and a continuous span, the connection details between the floor and beams are typically complicated, and in achieving structural simplicity, some voided slab systems without beams possess unobtrusive flexural strength. Presently, these problems need to be solved urgently by providing the technical and academic efforts demanded by the housing industrialization challenges in China.

**Figure 1:**
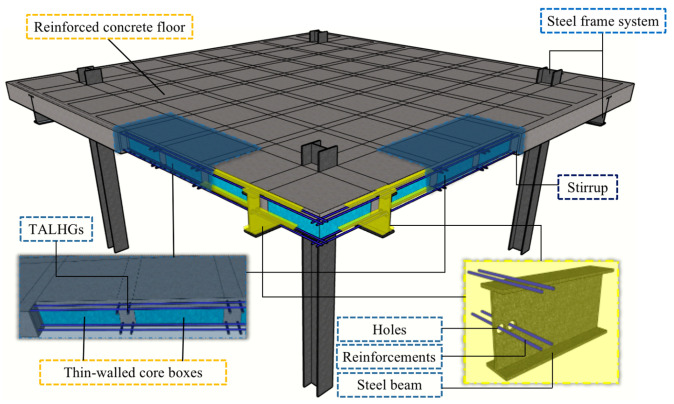
Conceptual configuration of the proposed system (thin-walled core boxes (TWCBs) hollow composite slab).

**Figure A1:**
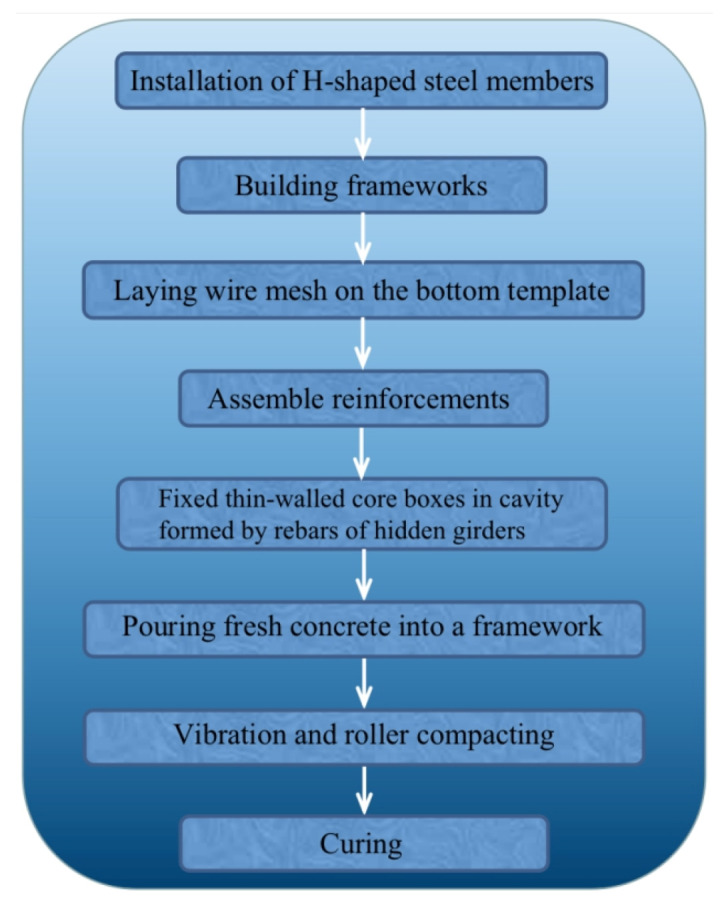
Procedure for the fabrication of such a composite slab.

**Figure A2:**
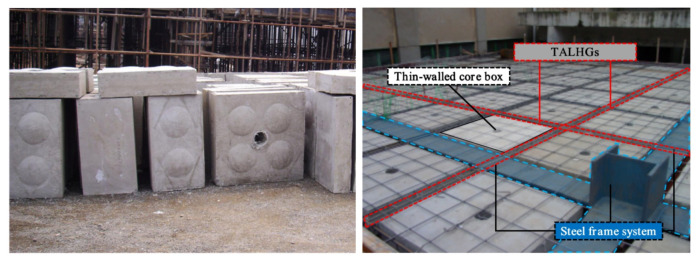
Photograph of fillers and proposed system.

A previous study has expounded on the flexural performance of a single steel–concrete composite beam with transverse and longitudinal hidden girders (TALHGs) [25]. However, the study does not address the application of such a system to an entire HCB, which has an inexplicit global mechanical behavior. Thus, regarding the issues mentioned earlier, a new long-span two-way lightweight steel–concrete composite slab voided with thin-walled core boxes (TWCBs), making it a hollow composite slab, is proposed in this study. The fabrication procedure is illustrated in Appendix A Figure A1. As shown in Figure 1, the TWCBs (Figure A2) are designed as fillers, creating a concrete voided slab system reinforced with TALHGs. Displacing traditional shear connectors (e.g., studs), H-shaped steel beams were adopted as a lateral frame system, with the bottom longitudinal and transverse rebars of the ribbed beams passing through the holes of the steel girders, thus simplifying the connection details between the floor and beams. In essence, this structure reduces raw material consumption and shortens the construction period, as shear connectors are replaced by structural rebars and composite slabs voided with TWCBs. However, research on the TWCBs hollow composite slab is still in the early stages of scientific investigation, with only a few tests and no large-scale experiments. Despite these endeavors, designers with knowledge of this innovative floor system still desire additional research to generate technical guidelines and convince end users.

For reinforced concrete slabs, the yield line method and strip method (based on the assumption of perfectly plastic material) are robust solutions for predicting the load-carrying capacity in bending. These two methods have proliferated into the wider structural engineering community because they are suitable for hand calculations [26,27]. The yield line method analysis starts with an assumed collapse mechanism and is then executed using virtual work and an equilibrium method to obtain the final solution: an upper-bound result [28]. Conversely, the strip method assumes zero torsional moments and is a lower-bound theorem. Although these methods have many advantages in engineering projects, many experimental tests indicate that the membrane effect may occur when slabs are thinner relative to the overall dimensions and possess the necessary horizontal edge restraint, thereby strengthening the ultimate load-carrying capacity [29,30,31,32,33]. At present, there are few studies on predicting the load-bearing capacity of a reinforced concrete hollow core slab using the yield line method, and almost no study has adopted an analysis that incorporates membrane action to calculate the ultimate load of the slab [34,35].

Consequently, this study investigates the flexural performance and design guidance for a TWCBs hollow composite slab via a large-scale experiment and theoretical analysis. First, a large-scale model of the composite slab was placed under uniformly increasingly distributed load to monitor crack growth, load-displacement, load–strain relationships, and strain profiles in typical locations. After experimental detection, a finite element model was constructed to validate the flexural deformation of the test specimen, and a practical and robust formula for predicting the yield load value was computed based on limit analysis using the upper-bound method. Furthermore, we propose a computation formula for bearing capacity that considers the membrane effect of the TWCBs floor. The results of this study provide a deeper understanding and exciting insights into the underlying performance of steel–concrete composite slab voided with the TWCBs system and will boost the application of composite structural systems in future construction toward achieving China’s long-term sustainable development goals.

## 2. Experimental Methods

### 2.1. General

**Figure 2:**
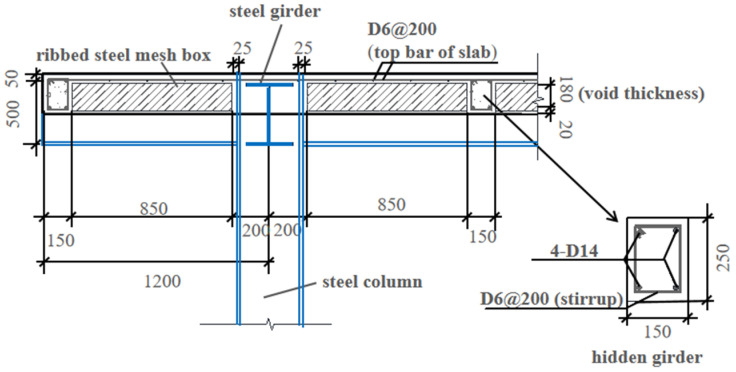
Typical cross-sectional details of the composite slab (units: mm).

A large-scale specimen was designed and fabricated, steel–concrete composite slab voided with thin-walled core boxes (F-TWCBs), with configurations presented in Table 1. The typical cross-sectional details of the composite slab are shown in Figure 2. Based on *t_s_* and void thickness, the number of fillers placed in the cross-section was determined, and the horizontal arrangement of voids was fixed when taking the spacing and width of hidden girders into account. All top and bottom flanges of the slab that enhanced voids’ durability were designed to be 50 mm and 20 mm, respectively.

### 2.2. Specimen

**Figure 3:**
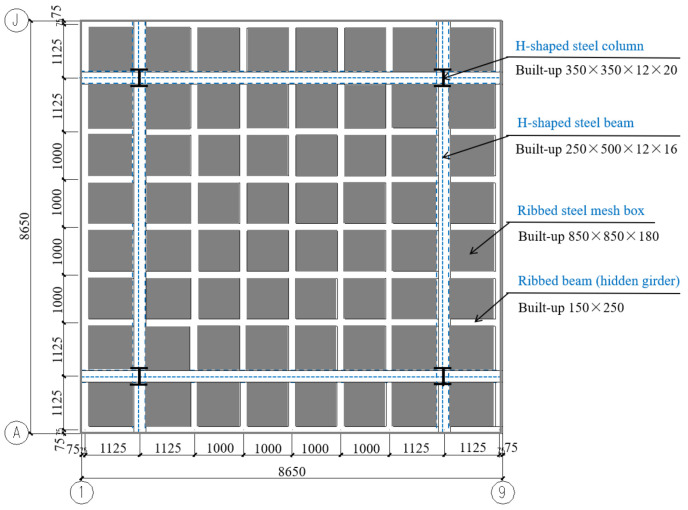
General layout plan (units: mm).

As shown in Figure 3, F-TWCBs was a composite slab voided with thin-walled core boxes, composed of hidden girders and cast-in-situ slab with lateral and vertical restraints of steel frame beams and columns, respectively. Four cantilevered plates (1200 mm in width), with the same cross-sectional details as the test piece, were set around the frame system to prevent excessive torsion and instability (Figure 3).

### 2.3. Material Properties

In the test, F-TWCBs was manufactured using concrete with a specified compressive strength of 23.4 Mpa, which exceeded the 16.7 Mpa suggested by JGJ/T 268-2012 [36]. During the pouring process, six standard cubes (150 × 150 × 150 mm) were produced following the standard test method [37] and cured under the same condition as the specimen. The compressive strengths and elastic modulus of six cubes measured at 28 days met requirements for performance on building concrete testing as set out in the standard GB/T 50081-2002 [37] (Table 2). The nominal yield stresses of reinforcing bars were 335 Mpa (longitudinal and transverse reinforcements) and 300 Mpa (stirrups). Table 3 summarizes the mechanical properties of the reinforcing bars. For all samples, yield stress, ultimate stress, and elastic modulus satisfied the standard requirements [38].

### 2.4. Instrumentation and Testing Procedure

**Figure A3:**
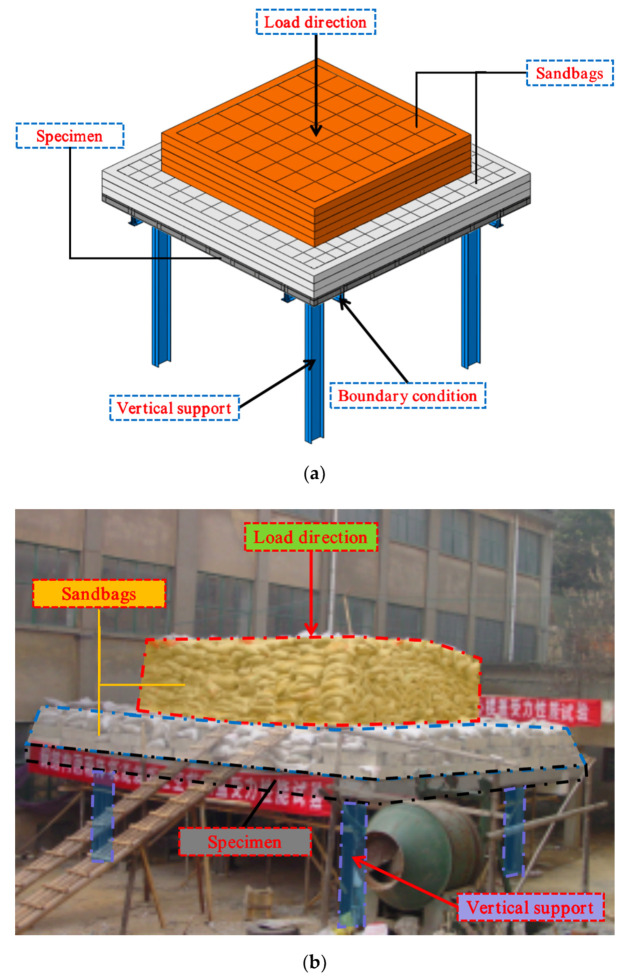
Loading system: (**a**) concept of the test setup; (**b**) photograph of the test setup.

Figure A3 illustrates the concept and a photograph of the test setup. The specimen was an orthotropic square two-way slab with all sides fixed by steel–concrete composite beam and supported vertically by H-shaped steel columns, respectively. In addressing the problem that it was inconvenient to load with indoor instruments (e.g., hydraulic jack and universal testing machine) due to the large loading area, sandbags full of sand (i.e., loading material) were distributed hierarchically on the surface of the test piece to simulate static uniform load.

**Figure 4:**
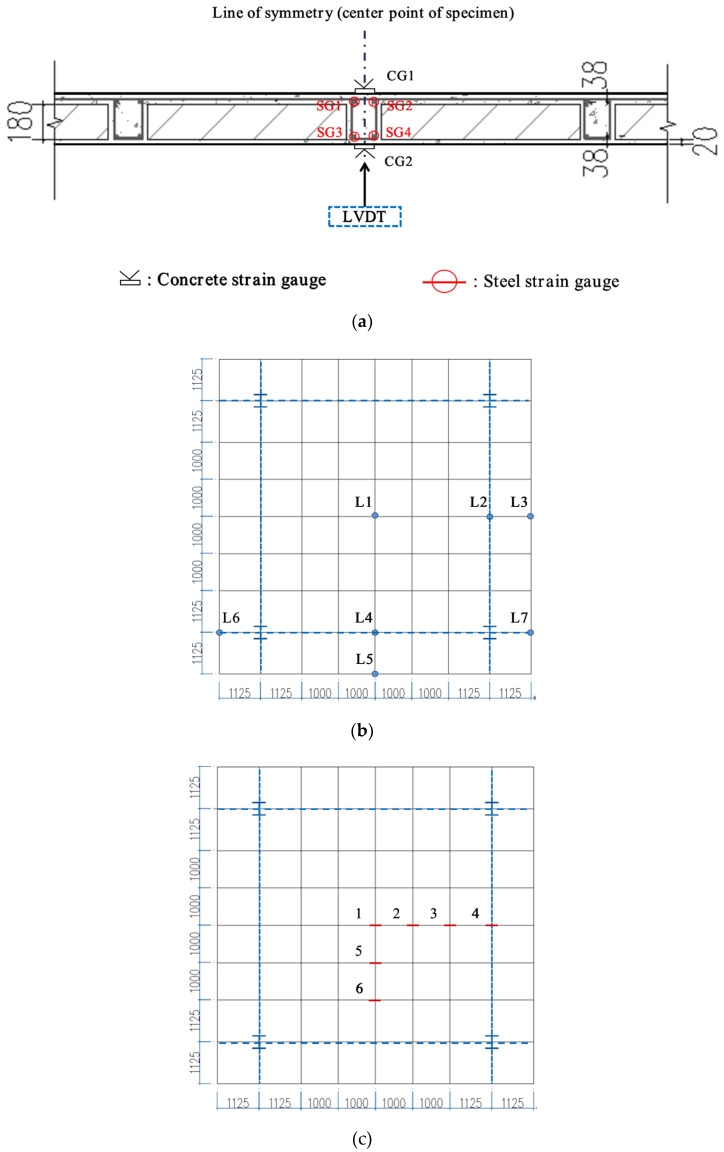
Measurement system: (**a**) detailed locations of strain gauges and the displacement sensor; (**b**) distribution of linear variable differential transducers (LVDTs); (**c**) distribution of strain gauges (units: mm).

Multiple linear variable differential transducers (LVDTs) (DH821-50, Wuhan Youtai Electronic Technology Co., Ltd., Wuhan, China) that monitored deflection of the specimen during the test were arranged across half spans of the slab in two directions (i.e., quarter area of the slab) owing to the symmetry of the structure (Figure 4b). Transducer L1 was used to measure vertical displacement at the center of the slab, LVDTs 3 and 5 below the mid-span of cantilevered plate edges, and remaining transducers along the span of the steel beams. Also, strain gauges (DH3818, Jingjiang Donghua Testing Technology Development Company, Jingjiang, China) were set up to examine the load-resistance characteristics of the slab system (Figure 4c). At each position, the specific locations of strain gauges and the displacement sensor (LVDT) refer to Figure 4a.

**Figure 5:**
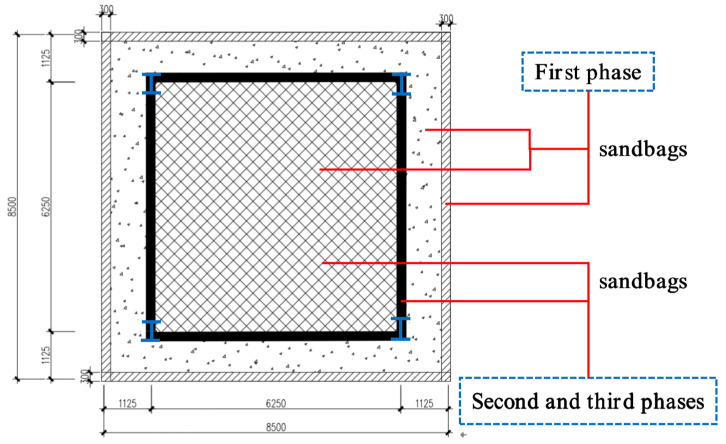
Loading phases of F-TWCBs (units: mm).

In a bid to inspect the development of cracks, the monotonic loading was applied discretely over 3 phases with breaks in-between (Figure 5). During the first phase, the test was conducted using a graded force control method performed with a load increment per stage of 1 kN/m^2^ until the mid-span strain of frame beam changed from negative to positive. At this time, the load on F-TWCBs with cantilevered plate was 13 kN/m^2^. In the following phase, the load increment per stage was switched to 2 kN/m^2^. On account of the composite beams being balanced, the cantilevered plate maintained the load value of the previous stage at the current phase, 13 kN/m^2^. Since over 82% ultimate load, a significant number of through-width cracks at ribbed beams have been observed, thus, the third phase was added to F-TWCBs with a load increment the same as the beginning phase.

## 3. Experimental Results and Discussion

### 3.1. Experimental Observations

**Figure 6:**
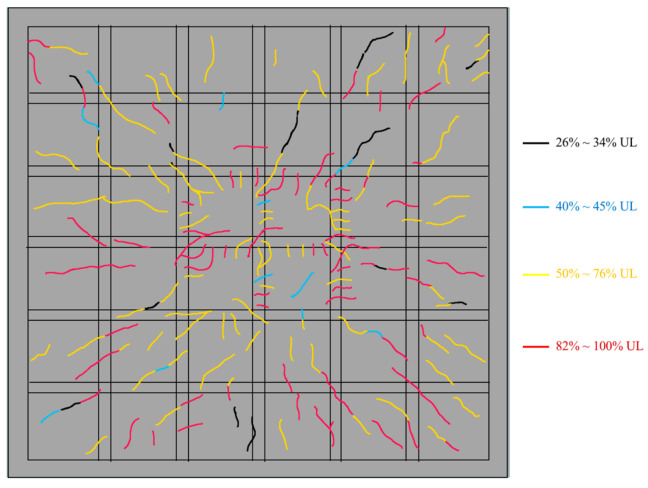
Crack propagation of the slab during loading (bottom surface).

As depicted in Figure 6, the specimen exhibited similar behavior to the reinforced concrete solid square slab. Before cracking, the test piece was in an elastic working state, and the load-displacement relationships were close to linear. When the uniformly distributed load on the slab reached 7 kN/m^2^, the concrete at the center of the slab began to crack. At this time, the measured maximum tensile strain of concrete was 71 × 10^−6^, just more than 70 × 10^−6^ obtained from the material test. The four corners at the bottom surface of the specimen began to show inclined cracks until 34% ultimate load (UL), which formed 45-degree angles to the steel girders. Subsequently, narrow cracks gradually occurred in the TWCBs area but did not extend to the ribbed beams. When the load achieved between 50% and 76% ultimate load, the bending crack propagation tended to expand from the periphery to the center as the yield line behavior of a typical two-way slab. Concurrently, through-width cracks of hidden girders in the middle region of the floor appeared in a slightly elevated number. After final loading, the occurrence of maximum crack width, 0.25 mm, was situated at the center point of the specimen, with the constant development of flexural cracks, which passed through adjacent hidden girders from voids. This phenomenon led to the asymmetrically intensive appearance of cracks in the central area of the slab. Eventually, the midpoint of the piece was concave, shaped like a bowl, meanwhile, whose cracks at the undersurface formed plastic strands along the diagonal. These observations indicated that when the ultimate bearing capacity is reached, the failure mode of the specimen is similar to that of the solid two-way slab, which belongs to bidirectional bending failure, and no shear failure occurs.

### 3.2. Load-Deflection Relationships

**Figure 7:**
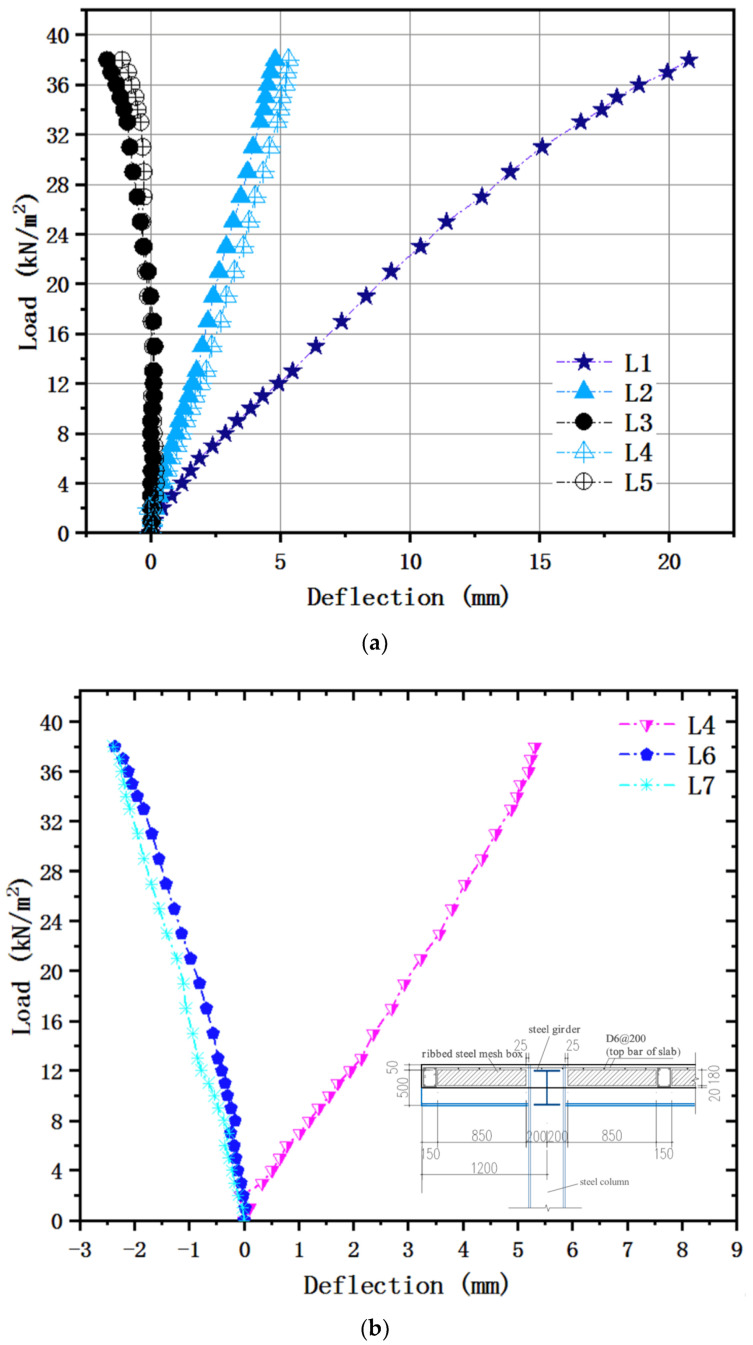
Load-deflection relationships: (**a**) bidirectional locations of F-TWCBs; (**b**) steel–concrete composite beam of F-TWCBs.

The relationships between the load and corresponding deflection in bidirectional locations for the test piece are illustrated in Figure 7a. According to the experimental data and phenomenon, the emphasis needed to remain on existing a minor inflection at the load of 7 kN/m^2^ in the curve, followed by a smooth elastic–plastic transition without a specific limit of linear behavior. As for the fact mentioned earlier about F-TWCBs, it can be attributed to the particular structural form of the steel–concrete composite hollow floor, whose stiffness is diminished slowly with a smattering of new tension cracks. Thus, the load increases linearly with the displacement. The test ended when the load reached 38 kN/m^2^, and the maximum vertical displacement at the center of the slab was 20.76 mm, around 1/3322 of its span, suggesting that the specimen has a large deformation capacity. Meanwhile, this value is less than the floor displacement limit of 25 mm (l/250) specified in GB 50010-2010 [39], indicating that the bending stiffness of the slab meets the requirement of the specification. The comparison of deflections in vertical directions (i.e., L2 and L3 versus L4 and L5, respectively) revealed that the various trends were almost indistinguishable, although there were a few more different values. So, it can be reasoned that the specimen is an approximately isotropic plate possessing resemble stiffness in the X and Y directions. Besides, the deflections at points L3 and L5 became negative from the load of 19 kN/m^2^, because as the load increased, the mid-span of the slab was concave, and the four corners rose. In practical engineering, peripheries of the slab connect to frame beams as a whole; there are walls on it. The outer boundaries are open to more energetic constraints, causing the displacement to be smaller than the experimental value. Figure 7b compares the load with girder flexibility recorded under cumulatively external loads, where the maximum deflection for F-TWCBs (5.313 mm) was measured as merely one-thousandth of the effective length, far less than the provision of the Chinese specification (one-fiftieth of the span) [40]. Hence the composite beam does not even enter the yield step, further showing the inexistence of the failure stage in the curve. Notwithstanding the flange of the frame beam was not welded with shear connectors, whose overall performance could still fulfill the essentials of regular employment, since the steel component and concrete slab behaved as a unitary part, in that event, the bottom bars of hidden girders acted as shear connectors maintained complete elasticity. Counting these, the boundary condition of F-TWCBs is relatively rigid, it can be preliminarily inferred.

### 3.3. Load–Strain Curves and Strain Profiles

**Figure 8:**
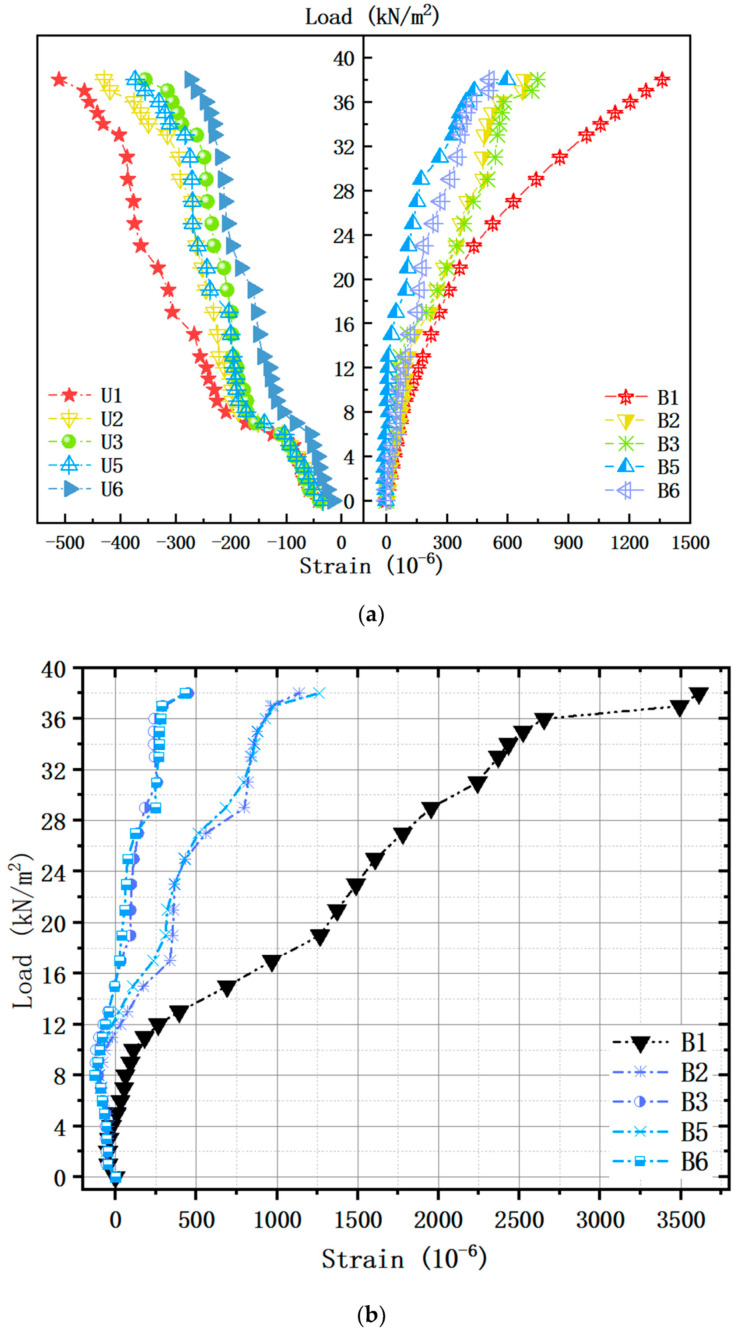
Load–strain curves in bidirectional locations: (**a**) concrete of F-TWCBs; (**b**) rebars of F-TWCBs.

Load–strain curves for the specimen are illustrated in Figure 8. In the name of the lines, the first symbol indicates a detailed location in the concrete section (U = upper and B = bottom). In order from the upper side, the strains were measured at the top surface of the concrete (CG1), top bars (SG1 and SG2), bottom bars (SG3 and SG4), and the bottom surface of the concrete (CG2) (refer to Figure 4a). During the analysis, the results of rebars measured in two locations of each height were averaged (Figure 8b). As for the distribution of internal forces within the concrete, the bottom surface was basically in a tension state, and the upper surface in a compressive state, identical with that of the solid slab (Figure 8a). Moreover, the conditions and trends of concrete strain for the specimen in X and Y directions were primarily consistent, and the tensile strain at the center point was the largest and decreased with the addition of the distance from the mid-span. Judging from the absolute value of the flexural stiffness of the section *B_s_* (calculated using Equations (1) and (2)) (Table 4), we discovered the measured ones at the bidirectional symmetrical position of the slab were close, uniform with the macroscopic deflection analysis in the last section.

(1) $$B_{s}=\frac{M}{1/\rho }$$

(2) $$\frac{1}{\rho }=\frac{{\varepsilon^{\prime }}_{s}+\varepsilon_{s}}{h_{0}-{a^{\prime }}_{s}}$$

where M, $\frac{1}{\rho }$, and $h_{0}$, respectively, are the bending moment, curvature, and effective height of section; ${a^{\prime }}_{s}$ is the distance from the centerline of upper reinforcement to top surface; ${\varepsilon^{\prime }}_{s}$ and $\varepsilon_{s}$, respectively, are the measured strains of upper and bottom rebar in the same location; the flexural stiffness of the section was estimated by considering the measured strains of rebars when the external load was 6 kN/m^2^ (before cracking). Therefore, the check calculation of deformation can be carried out according to the isotropic two-way plate in the engineering design. For F-TWCBs, the strain values of the lower reinforcements at B2, B3, B5, and B6, were negative before the specific uniformly distributed loads (11 kN/m^2^, 15 kN/m^2^, 12 kN/m^2^, and 15 kN/m^2^, respectively), including that in the central location. This fact was because the stiffness of the hidden girders and the ultimate bearing capacity of the frame beams were a great deal higher than the rest in this trial, which could effectively prevent the lateral deformation of the slab, generate membrane pressure, and lessen the mid-span bending moment and cracks. According to the test results of material properties, the average yield strain of longitudinal rebars was 1837 × 10^−6^. If the material exceeds this benchmark, it will come under plastic deformation. Thus, when the uniformly distributed load reached about 27 kN/m^2^, the measured tensile strain value of the bottom reinforcement of the ribbed beam at the center of the slab (B1) was 1779 × 10^−6^, indicating that the test piece is close to yielding. At this time, the measured maximum compressive strain (408 × 10^−6^) of the concrete at the upper surface of the slab was far less than its ultimate compressive strain. Then, when loaded to 29 kN/m^2^, the measured maximum tensile strain (1954 × 10^−6^) of the rebar arrived its yield strain. As the load increased, the cracks at the underside of the slab developed symmetrically and widened. It was worth noting that at 36 kN/m^2^, the measured maximum tensile strain of the rebar at B1 (2653 × 10^−6^) was very close to its ultimate strain value (2710 × 10^−6^). Hereafter, the strain growth of the reinforcement was much larger than that of any previous stage, and its final strain value was 3612 × 10^−6^ (Figure 8b).

**Figure 9:**
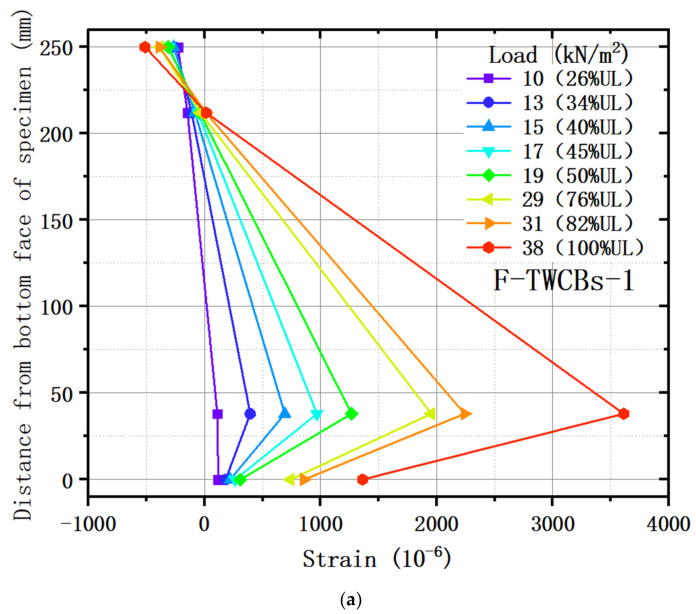

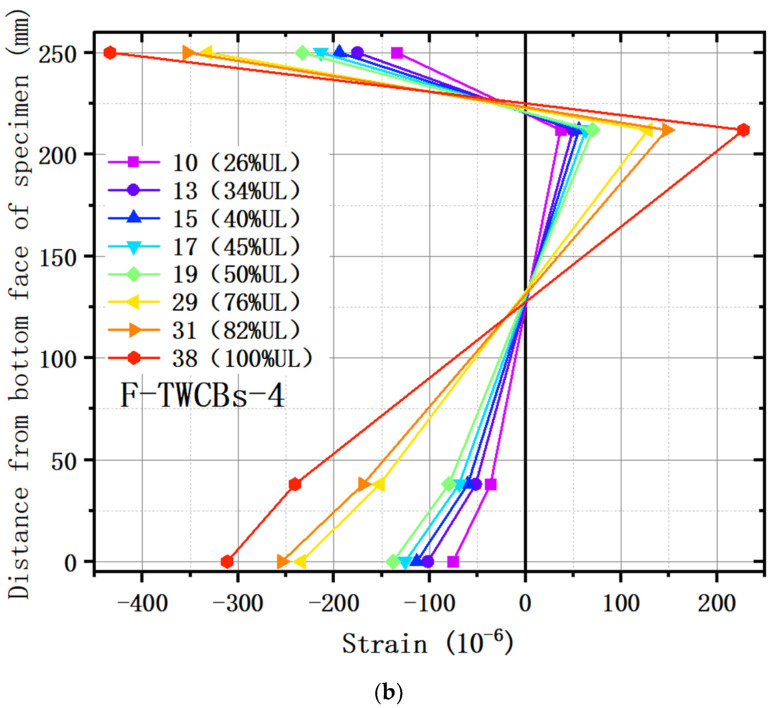
Strain profiles in locations: (**a**) center point of F-TWCBs; (**b**) mid-span of the steel–concrete composite beam for F-TWCBs.

The strain profiles of the test slab along the height are presented in Figure 9, where representative data were selected according to the loading condition of crack development. In essence, the neutral axis location was affected by the plasticity due to the flexural crack propagation and shifted upward with the increment of load. More specifically, the strains were linearly distributed along the cross-section at the center of the slab, up to approximately 0.26 UL. After that, the interfacial bond resistance between concrete and bottom rebars began to decrease, which significantly reduced the strain on the bottom surface of the specimen (Figure 9a). However, this fact did not affect the overall composite performance of the hollow slab, and the load-carrying capacity gradually increased before reaching UL. As viewed from the strain profile at the mid-span of the composite beam (Figure 9b), the lower flange concrete was under compression and well bonded together with the bottom reinforcement. Nevertheless, the upper flange concrete was not under tension because the load was piled up directly above the frame beam throughout the trial phases. In the terminal state of F-TWCB, the measured compressive strain value of the concrete at the upper surface of the mid-span frame girder was 434 × 10^−6^, and the tensile strain at the bottom was 312 × 10^−6^, both of them were far less than its cracking strain. Concurrently, the strain value of the upper steel bar (228 × 10^−6^) was positive, while that of the bottom one (241 × 10^−6^) was negative. This phenomenon was consistent with the behavior of the reinforced concrete floor slab when subjected to uniform loads and lateral restraints to generate compressive membrane forces [41]. Given this evidence, it can be deduced that the membrane effect of the test piece is worth considering.

## 4. Finite Element Analysis of Flexural Deformation

### 4.1. Finite Element Model

**Figure 10:**
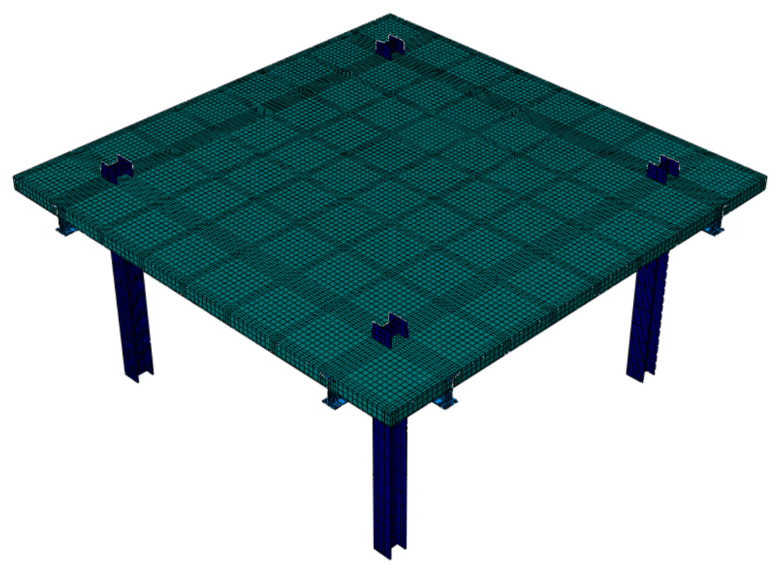
Finite element model (FEM) of test specimen F-TWCBs.

A finite element (FE) model of the tested steel–concrete composite slab was constructed using the FE software, ABAQUS 2019 (Dassault Systèmes Simulia Corp., Johnston, RI, USA). C3D8R solid elements, with linear reduced integration, were utilized to simulate the concrete slab, steel beams, and columns. T3D2 truss elements were applied to simulate the rebars. The material properties of concrete were modeled using a stress-strain curve with the average values listed in Table 2. The following values were used: compressive strength = 21.57 Mpa, elastic modulus = 30,800 Mpa, Poisson’s ratio = 0.2, and density = 2.4 × 10^3^ kg/m^3^. The mechanical properties of the steel were consistent with the average values listed in Table 3. Poisson’s ratio and the density of the steel used in the model were 0.285 and 7.85 × 10^3^ kg/cm^3^, respectively. The concrete and steel plasticity models from ABAQUS were based on data supplied by a prior study [42]. The normal contact points between the concrete slab and steel framework were defined as hard contacts, and the tangential contact points between them adopted an isotropic friction formulation called the penalty. *Tie* constraints were applied to simulate the welding relationship at the interface between the steel beams and the columns. In addition, we set the rebars as an embedded region of the concrete slab to simulate its actual state. Under static loading, the column foot was linked to the foundation and buried in the earth; and it can be considered that no displacement occurred. Thus, the model uses a completely fixed method to constrain the bottom surface of the column. To reduce the cost of the computer calculation while ensuring the accuracy of the simulation, we divided the model into regions using the structured grid and sweep grid technologies, with a mesh size of 100 mm × 100 mm (Figure 10).

### 4.2. Finite Element Results and Discussion

**Figure 11:**
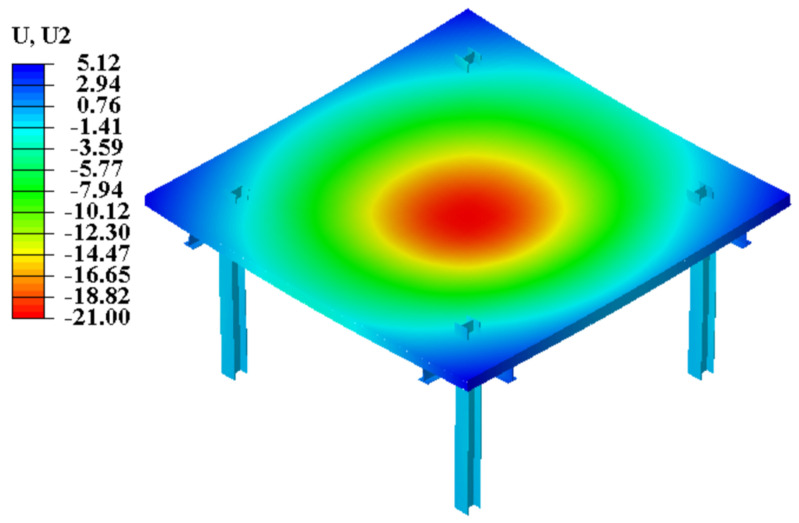
Flexural deformation of finite element (FE) analysis.

**Figure 12:**
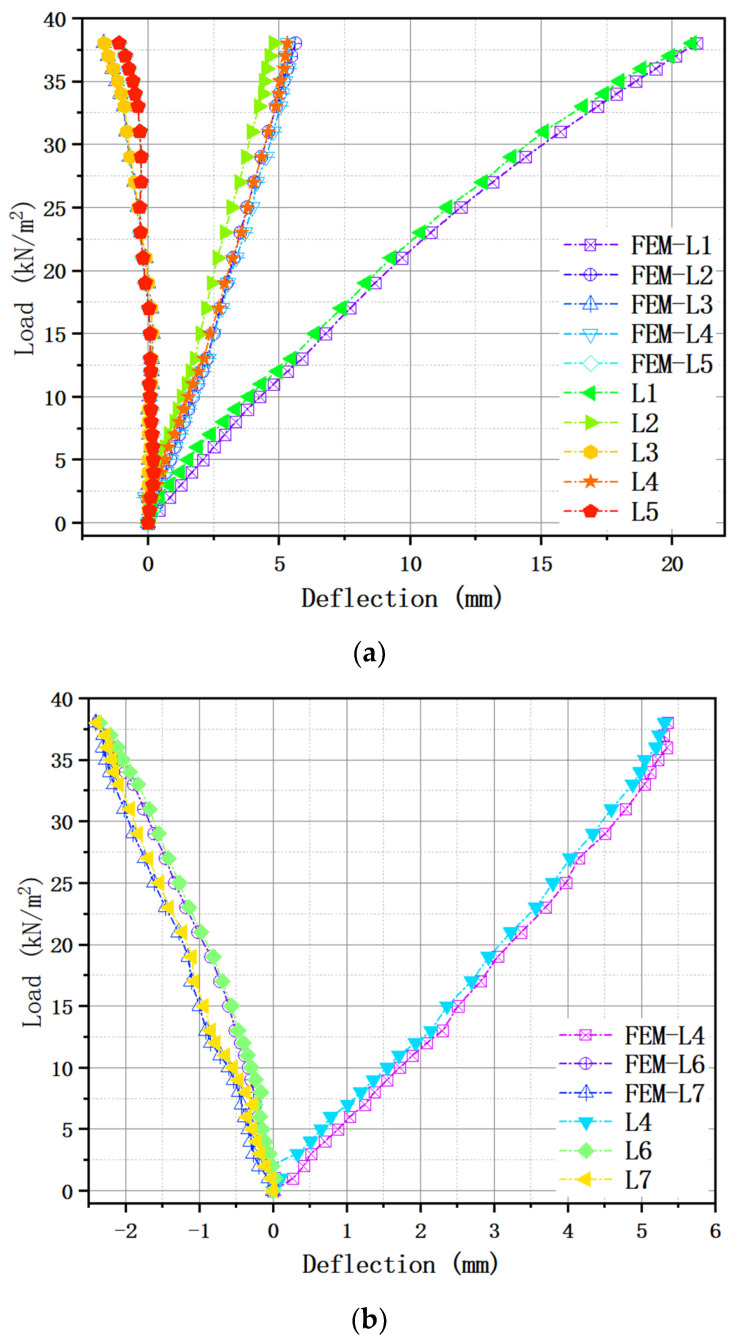
Comparison of the load-deflection relationships between the FEM and the test specimen: (**a**) bidirectional locations of F-TWCBs; (**b**) steel–concrete composite beam of F-TWCBs.

Figure 11 presents the final deformation result of the finite element model (FEM), which has a failure mode and maximum deflection that correspond to the experimental detection results. The load-deflection curves for the FEM and the test specimen are presented in Figure 12. Based on this comparison, the FEM accurately simulates the initial flexural rigidity of the composite slab. The loading method in the simulation corresponded to that in the trial, and the central deflection of the F-TWCBs derived using the FEM (21 mm) is only 1% larger than that obtained in the experimental results (Figure 11). The maximum deflections of L3 and L5 in the FEM (−1.69 mm and −1.11 mm) are negative, validating the correctness of the slab edge ascending and the isotropic plate (Figure 12a). Furthermore, when the deflection values changed from positive to negative in the FEM, the load value (19 kN/m^2^) was the same as that from the test. Meanwhile, the mid-span deformations of the steel beams computed from the FEM were only 1–16% larger than that from experimental observations; the data on FEM-L6 and L7 confirm the phenomenon of the ends of steel beams being raised in a real-world situation (Figure 12b). Given these observations, the steel components and the concrete slab behave as a unitary part through their structural rebars, can also be inferred via finite element analysis.

## 5. Theoretical Analyses of Bearing Capacity

### 5.1. Predicted Equation Considering Limit Analysis Using the Upper-Bound Method

**Figure 13:**
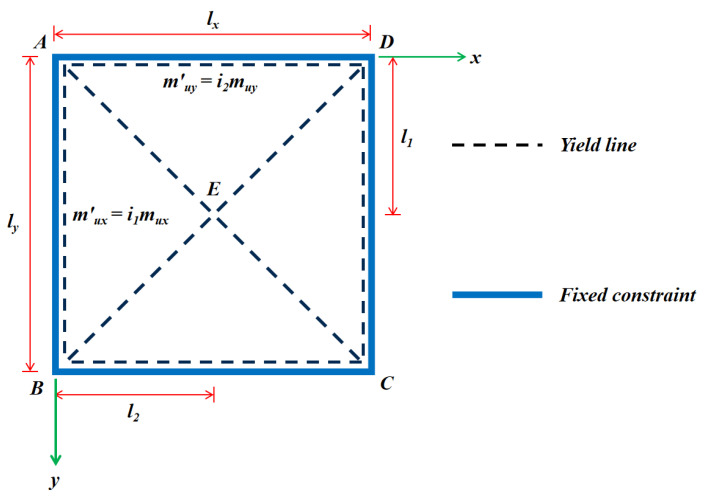
Yield line mode of a square slab with a fixed boundary condition on four sides. Notes: A–D are the four corner points of the slab; *l_x_* and *l_y_* are the width and length of the slab, respectively; *l_1_* and *l_2_* represent the distance between the x-axis and y-axis, respectively, from the intersection point of the yield lines in the slab.

The yield line method uses rigid plastic theory to compute the failure loads corresponding to the given plastic moment resistance in various parts of a slab and is a powerful method for predicting the failure load of reinforced concrete slabs [43,44,45,46]. The bearing capacity of the specimen estimated using the limit analysis of the upper-bound method is as follows:

Failure mechanisms such as the crack pattern and failure mode must be identified to calculate the ultimate load-bearing capacity of the specimen. In line with the description in the previous section, the specimen subjected to uniform load in this large-scale test is a square slab with fixed boundary conditions on four sides, whose failure mode (diagonally distributed yield line) is consistent with the traditional two-way cast-in-situ reinforced solid concrete floor (Figure 13). In this method, the predicted ultimate uniformly distributed load value ($q_{cal}$) is calculated using Equation (3) [41].

(3) $$q_{cal}=\frac{6m_{uy}\mu Y^{2}}{l_{y}{}^{2}{\left(l_{y}/l_{x}\right)}^{2}{\left\{{\left[{\left(X/Y\right)}^{2}+3\mu {\left(l_{y}/l_{x}\right)}^{2}\right]}^{1/2}-\left(X/Y\right)\right\}}^{2}}$$

where *l_x_* and *l_y_*, respectively, are the breadth and length of the floor; μ is the orthotropic coefficient, $\mu =m_{uy}/m_{ux}$; $m_{ux}$ and $m_{uy}$, respectively, represent the positive ultimate resistance moment per unit width in the X and Y directions at the edge of the slab. The assumed parameters X and Y are determined using $2\sqrt{1+i_{1}}$ and $2\sqrt{1+i_{2}}$, where i is the negative ultimate resistance moment per unit width (${m^{\prime }}_{ux}$ or ${m^{\prime }}_{uy}$) versus the positive ultimate resistance moment per unit width ($m_{ux}$ or $m_{uy}$) in the corresponding direction.

**Figure 14:**
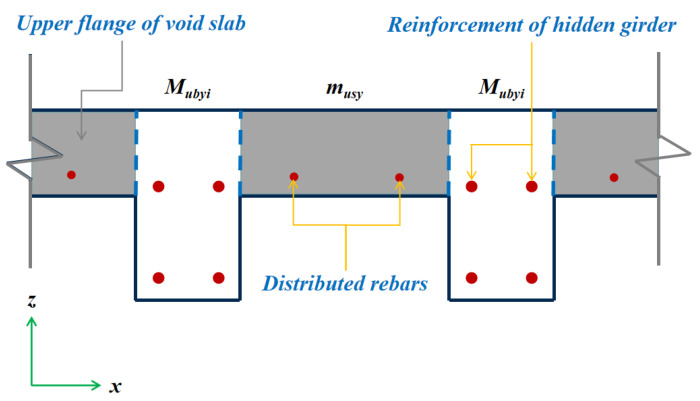
Equivalent section of voids slab. Notes: $M_{ubyi}$ expresses the ultimate resistance moment of a single hidden girder in the Y direction, and $m_{usy}$ represents the ultimate resistance moment per unit width of the upper flange of a voided slab.

Regarding the solution of $m_{ux}$ and $m_{uy}$ (taking the cross-section of the z-x plane as an instance), the voided slab is regarded as a multi-ribbed floor, with the sum of the positive resistance moment equally distributed along its length (Figure 14). The ultimate resistance moment of a single hidden girder in the Y direction ($M_{ubyi}$) and ultimate resistance moment per unit width of the upper flange of a voided slab ($m_{usy}$) are computed using the current concrete design code [39]. Hence, $m_{uy}$ and $m_{ux}$ can be obtained as follows:

(4) $$m_{uy}=\frac{m_{usy}l_{x}+\sum_{i=1}^{n}M_{ubyi}}{l_{x}}$$

where *n* is the number of ribbed beams parallel to the Y direction.

Using Equation (3), the ultimate load-bearing capacity of the specimen ($q_{cal}$) was calculated to be 20.2 kN/m^2^, approximately half the value of the result obtained from the test (38 kN/m^2^). Although the prediction using this method has a sufficient safety margin, the outcome is too conservative. Therefore, a more pragmatic approach for an accurate prediction of the ultimate load-bearing capacity should be utilized.

### 5.2. Calculation Formula Incorporating the Membrane Effect

An accurate solution that addresses the conservative result of the preceding method can be obtained by embodying the membrane effect. If the rigid boundary condition constrains the lateral displacement of the floor, the trend of the slab edge moving outwards will react with the frame girder when its geometry changes, such that the compressive membrane force will emerge in the plane and dramatically improves the ultimate bearing capacity [41]. To analyze such a novel slab, the following assumptions (based on membrane action) are made:

(1) The yield line mode of the specimen is diagonal (Figure 13).
(2) The cross-section of the floor in a single direction is divided into several I-sections because the fillers and the cast-in-situ concrete ribbed beams can work in concert. Ribbed strips along the X and Y directions constitute a slab and are straight in the yield section, with the sum of the elastic strain, creep strain, axial shrinkage strain ε, and lateral displacement t, are taken as 0.
(3) The concrete in the compression zone reached its yield strength.
(4) When the center deflection δ is 0.5 times the thickness (i.e., 125 mm), the component achieves the ultimate load [41].

**Figure 15:**
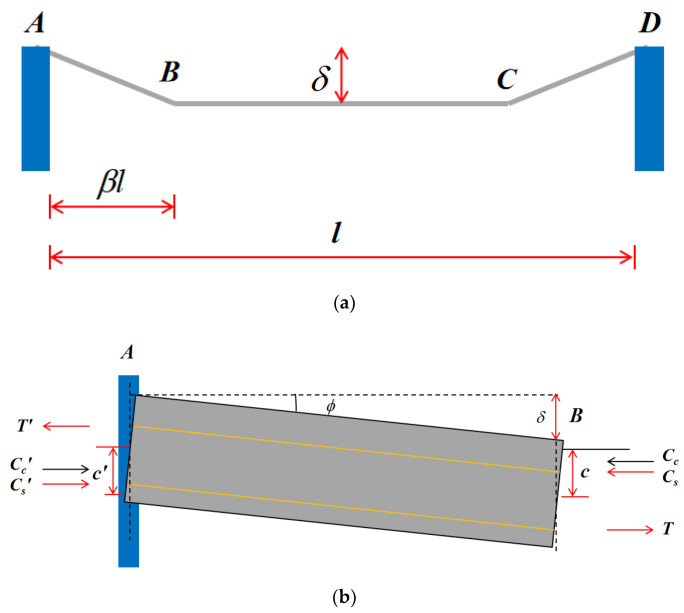
Single ribbed strip in general: (**a**) deformation in one direction; (**b**) stress of section between yield sections A and B. Notes: A and D are the two endpoints of the single ribbed strip; B and C represent the two intersection points of the yield lines in a two-way slab.

First, verifying the deformation and stress of a single ribbed strip is generally essential (Figure 15). Thus, the distance between point A and point B expressed using displacement geometry can be obtained as follows:

(5) $$\left[\beta l+0.5\varepsilon \left(1-2\beta \right)l+t\right]\sec\varphi =\left(h-c^{\prime }\right)\tan\varphi +\left(1-\varepsilon \right)\beta l-c\tan\varphi$$

where l, φ, and h are the length, slope, and thickness of the cross-section, respectively; βl represents the initial length between points A and B; $c^{\prime }$ and c are the heights of the compressed area in sections A and B, respectively. Because φ and ε are very small in real-world situations, the following formulas can be derived:

(6) $$\sin\varphi =2\sin\frac{\varphi }{2}=\frac{\delta }{\beta l}$$

(7) cosφ=1

Furthermore, the short-term effect was considered in the experiment, i.e., ε=0 and t=0. Thus, Equation (5) can be simplified to:

(8) $$c^{\prime }+c=h-\frac{\delta }{2}$$

Concurrently, to balance the force system, the membrane forces acting on sections A and B are equal:

(9) $${C^{\prime }}_{c}+{C^{\prime }}_{s}-T\prime =C_{c}+C_{s}-T$$

where ${C^{\prime }}_{c}$ and $C_{c}$ are the concrete pressures acting on sections A and B, respectively. Similarly, ${C^{\prime }}_{s}$ and $C_{s}$ represent reinforcement pressure while $T^{\prime }$ and T represent tension on sections A and B, respectively.

**Figure 16:**
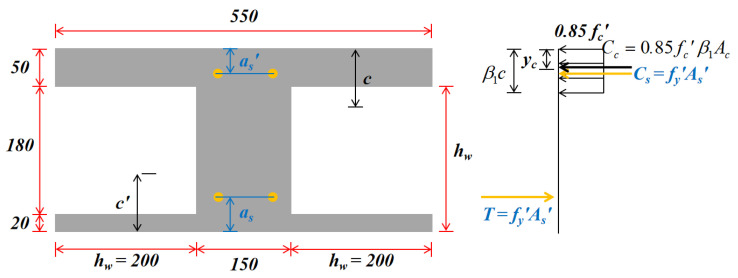
The stress distribution at the ultimate strength of the ‘I’ type equivalent cross-section for F-TWCBs (units: mm).

The width of the flange and the stress distribution at the ultimate strength of the ‘I’ type equivalent cross-section for F-TWCBs were selected based on the ACI318 design code [47] (Figure 16). c and $c^{\prime }$ can then be calculated by substituting the dimension parameters and material properties of the section into Equation (9) and combining the results with Equation (8). In addition, the action points of concrete in the compression zone of the two sections can be obtained. For positive moment sections, the resultant forces of stress at the section, $C_{c}$, $C_{s}$, T, and the membrane force $n_{u}$ acting on the thickness centerline are all in a static equilibrium state. Meanwhile, the point resistance moment of these forces to the centerline of the slab is $m_{u}$. Thus, the balanced equation for a strip of unit width is as follows:

(10) $$n_{u}=C_{c}+C_{s}-T$$

(11) $$m_{u}=C_{c}\left(h/2-y_{c}\right)+C_{s}\left(h/2-{a^{\prime }}_{s}\right)-T\left(h/2-a_{s}\right)$$

where $y_{c}$ is the distance from the action point of the compressional concrete to the edge of the slab; $a_{s}$ and ${a^{\prime }}_{s}$ represent the distance between the center of tensile/compression rebars and the lower/upper surface of the concrete, respectively. In the yield section (A) of a negative bending moment, the expression $M_{u}$ is similar to Equation (11) and ${n^{\prime }}_{u}=n_{u}$ can be produced from the balanced formula for force.

Based on the above analysis, the sum of the moment taken by the resultant force $n_{u}$ and the point resistant moment $m_{u}$ at the yield section of the positive moment to the fixed section is as follows:

(12) $$M=C_{c}\left(h/2-y_{c}\right)+{C^{\prime }}_{c}\left(h/2-{y^{\prime }}_{c}\right)+4T\left(h/2-a_{s}\right)-0.85{f^{\prime }}_{c}\beta_{1}A_{c}\delta$$

where ${y^{\prime }}_{c}$ is the distance from the action point of the concrete in the compression zone to the edge of the slab; $\beta_{1}$ is the ratio of the height of the equivalent rectangular stress diagram to the height of the neutral axis, with a value of 0.85 based on the ACI318 design code [47]; $A_{c}$ is the height of the compression concrete zone; ${A^{\prime }}_{s}$ is the section area of the compression reinforcement. If the AB or CD portion of the strip produces a virtual angle θ, the virtual work formula for the forces at the yield section can be obtained:

(13) $$M\theta =\left(m_{u}+{m^{\prime }}_{u}-n_{u}\delta \right)\theta$$

According to the energy conservation principle, external work and internal work should be equal. Therefore, the value of Equation (13) matches the work done by the load when the strip undergoes the displacement. Consequently, the ultimate uniformly bearing capacity of a single ribbed strip in a unit width can be computed.

Finally, the preceding analyses are employed for calculations on the entire slab. It is assumed that the central location of the floor produces a virtual unit displacement in the direction of the load. Thus, the virtual work equation is as follows:

(14) $$\iint qd_{x}d_{y}=2\int_{0}^{l_{y}}\left(m_{ux}+{m^{\prime }}_{ux}-n_{ux}\delta \right)\frac{2}{l_{y}}dy+2\int_{0}^{l_{x}}\left(m_{uy}+{m^{\prime }}_{uy}-n_{uy}\delta \right)\frac{2}{l_{x}}dx$$

With reference to the particularity of the structure, the calculation form of the integral is the sum of the work done by the ribbed strip in different positions. Thus, the final calculation formula for the ultimate uniformly distributed load can be computed as follows:

(15) $$\frac{q_{u}}{3}l^{2}=\frac{8}{l}\left[C_{c}\left(h/2-y_{c}\right)+{C^{\prime }}_{c}\left(h/2-{y^{\prime }}_{c}\right)+4T\left(h/2-a_{s}\right)\right]\times 5-\frac{8}{l}C_{c}\left(3\delta \right)$$

Substituting the known parameters of F-TWCBs into Equation (15), $q_{u}$ is calculated as 39.9 kN/m^2^, close to the value in the large-scale experiment, 38 kN/m^2^. However, the value obtained using the prediction method in Section 5.1 was 20.2 kN/m^2^, approximately 50% lower than the value estimated using the calculation formula incorporating membrane effect. Consequently, this proves that the membrane effect provides a considerable safety margin for the specimen, and the original steel–concrete composite beams serve as strong boundary constraints. The resulting improvement in load-bearing capacity should be considered in future designs.

## 6. Conclusions

This study presented an experimental work of a new TWCBs hollow composite slab and investigated its flexural performance by preparing and testing a large-scale composite slab subjected to uniformly distributed loads. The theoretical analyses for predicting the load-bearing capacity of the specimen were carried out based on limit analysis using the upper-bound method and considering the membrane effect. The following conclusions were drawn from the results of this study:

(1) The specimen showed similar behavior to a two-way isotropic solid slab under bending. At the end of loading, the main flexural cracks passed through adjacent hidden girders from voids at the central area of the slab.
(2) In the experiment, the ultimate bearing capacity of the composite floor was approximately 38 kN/m^2^, with a maximum deflection of 20.76 mm. Although the flange of the steel–concrete composite beam was not welded with shear connectors, its deflection at the mid-span is far less than the limit value of GB/T 50152-2012 when the flexural member is damaged.
(3) Based on the load–strain relationships in bidirectional locations, it can be inferred that the specimen is an isotropic hollow slab. In the final stage of the trial, the strain value of upper rebars at the mid-span of the frame girder was positive while that of bottom rebars was negative, consistent with the behavior of the uniformly loaded and laterally constrained reinforced concrete slab when the compressive membrane force is generated.
(4) The predicted equation considering limit analysis using the upper-bound method provided a conservative result in estimating the flexural strength of the specimen, where the ratio of experimental ultimate uniformly distributed load value to that of computation was 1.88.
(5) On the other hand, the proposed theoretical solution incorporating membrane effect was closer to the real datum; therefore, this method can reasonably be applied to calculate the load-bearing capacity of the steel–concrete hollow composite slab voided with thin-walled core boxes.
(6) The membrane effect will have a significant influence on the flexural strength of the hollow slab. However, this effect must be considered with caution, typically when the floor is surrounded with unreliable lateral restraints and configured inappropriate steel ratio in the cross-section.
(7) As established by the test results and valuation, the steel–concrete composite slab voided with thin-walled core boxes can replace a conventional overweight solid slab as a two-way slab.

This study offers both academic and practical contributions. First, the outcomes of this study will serve as a theoretical foundation for future researches of composite structural systems and advanced engineering structures. Moreover, the findings indicated that the novel composite slab performs better, contributing to significant savings in the structural material demand and building cost. It provides a benchmark for the practical design and application of composite structural systems in sustainable construction towards meeting China’s green building criteria and sustainable development agendas.

## Figures and Tables

**Table 1:** Configuration of the specimen.

Specimen	*t_s_* (mm)	Width (mm)	Length (mm)	Void Filler	Reinforcing Bar				
					Hidden Girder			Slab	
					Upper	Bottom	Stirrup	Upper	Bottom
F-TWCBs	250	6250	6250	Thin-walled core box	2-D14 @100	2-D14 @100	D6 @200	D6 @200	-

*t_s_*: the thickness of the slab. D: diameter of rebars. @: spacing of rebars.

**Table 2:** Mechanical properties of concrete.

Sample	Age (d)	Compressive Strength (Mpa)	Elastic Modulus (Mpa)	Applied Specimen
1	28	21.57	30,800	F-TWCBs

Note: Values were averaged from the six cubes.

**Table 3:** Mechanical properties of steel reinforcement.

Diameter (mm)	Yield Stress (Mpa)	Ultimate Stress (Mpa)	Elastic Modulus (Mpa)	Applied Specimen
6	326.67	456.86	2.03 × 10^5^	F-TWCBs F-TWCBs
14	358.26	528.38	1.95 × 10^5^	

Note: Values were averaged from three coupons for bar samples.

**Table 4:** Measured strains of rebars in bidirectional locations and corresponding sectional bending stiffness of the specimen.

Bottom Reinforcing Bar		Upper Reinforcing Bar		*B_s_* (N·mm^2^)
Location	Strain (×10^−6^)	Location	Strain (×10^−6^)	
B2	−72.75	U2	−32.21	7.05 × 10^13^
B5	−78.14	U5	−31.73	6.71 × 10^13^
B3	−78.93	U3	−8.17	2.79 × 10^13^
B6	−82.63	U6	−11.06	2.59 × 10^13^

Note: the flexural stiffness of the section was estimated by considering the measured strains of rebars when the external load was 6 kN/m^2^ (before cracking).

## Abbreviation

F-TWCBs: steel–concrete composite slab voided with thin-walled core boxes.
